# Twelve-month incidence and clearance of oral HPV infection in HIV-negative and HIV-infected men who have sex with men: the H2M cohort study

**DOI:** 10.1186/s12879-014-0668-z

**Published:** 2014-12-31

**Authors:** Fleur van Aar, Sofie H Mooij, Marianne AB van der Sande, Chris JLM Meijer, Audrey J King, Dominique WM Verhagen, Titia Heijman, Roel A Coutinho, Maarten F Schim van der Loeff

**Affiliations:** Centre for Infectious Disease Control, National Institute for Public Health and the Environment (Rijksinstituut voor Volksgezondheid en Milieu, RIVM), Bilthoven, 3720BA The Netherlands; Cluster of Infectious Diseases, Public Health Service of Amsterdam (GGD), Amsterdam, The Netherlands; Center for Infection and Immunity Amsterdam (CINIMA), Department of Internal Medicine, Academic Medical Center, Amsterdam, The Netherlands; Julius Center for Health Sciences and Primary Care, University Medical Center Utrecht, Utrecht, The Netherlands; Department of Pathology, Vrije Universiteit-University Medical Center (VUmc), Amsterdam, The Netherlands; Department of Internal Medicine, Jan van Goyen Medical Center, Amsterdam, The Netherlands

**Keywords:** Oral, HPV, Incidence, Clearance, HIV, MSM

## Abstract

**Background:**

Our aim was to compare the 12-month incidence and clearance of oral high-risk HPV infection between HIV-infected men who have sex with men (MSM) and HIV-negative MSM.

**Methods:**

MSM aged 18 years or older were recruited in Amsterdam, the Netherlands. Questionnaire data and oral-rinse and gargle samples were collected at baseline, and after 6 and 12 months. HPV DNA was genotyped using the SPF_10_-PCR & DEIA-LiPA_25_ system (version 1). Determinants of oral HPV incidence and clearance were explored using Cox and logistic regression analyses respectively.

**Results:**

433 HIV-negative and 290 HIV-infected MSM were included in these analyses. The median follow-up time per participant was 12 months (range 3–15). During follow-up, 114 incident oral high-risk HPV infections were observed. The incidence rate of HPV-16 was 3.5/1000 person-months (PM) in HIV-infected and 0.9/1000 PM in HIV-negative MSM (IRR 4.1; 95% CI 1.3-13.2). The incidence rates of other high-risk HPV types ranged between 1.3-3.5/1000 PM in HIV-infected and 0.0-1.1/1000 PM in HIV-negative MSM. In multivariable analyses, HIV infection (adjusted hazard ratio [aHR] 3.8; 95% CI 2.3-6.2) and a higher number of recent oral sex partners (aHR 2.4 for ≥8 partners compared to ≤2; 95% CI 1.4-4.2) were associated with HPV incidence. Of the 111 baseline oral high-risk infections, 59 (53.2%) were cleared. In multivariable analyses, only a higher number of recent oral sex partners was associated with HPV clearance (adjusted odds ratio 3.4 for ≥8 compared to ≤2 partners; 95% CI 1.3-9.0).

**Conclusions:**

The incidence rate of oral high-risk HPV infection was higher in HIV-infected MSM and in those with a higher number of recent oral sex partners. Just over half of the oral high-risk HPV infections at baseline were cleared after 12 months, with a higher likelihood of clearance among MSM reporting higher numbers of recent oral sex partners, but no difference by HIV status.

**Electronic supplementary material:**

The online version of this article (doi:10.1186/s12879-014-0668-z) contains supplementary material, which is available to authorized users.

## Background

Human Papillomavirus (HPV), in particular oral infection with high-risk HPV type 16, has recently been recognized as a main etiologic agent for a subset of head and neck cancers [1]. Although the prevalence of oral high-risk HPV infection is relatively low among the general population, with estimates around 3.5% [2], a much higher prevalence of up to 25% has been reported among HIV-infected men who have sex with men (MSM) [3],[4].

The natural history of oral HPV infection is not yet well understood. Previous studies suggest that the incidence rate of oral HPV infection is lower than that of anal and genital HPV infections [5]-[7], whereas the clearance rate seems similar or higher compared to genital and anal HPV infection [5],[6],[8]. Oral sex is thought to be the most important risk factor of prevalent oral HPV infection, but also male gender, increasing age, a higher number of recent or lifetime partners, current smoking and HIV-infection have often been associated with oral HPV infection [3],[4],[9],[10]. Factors that have been associated with incident and persistent infections differ across studies. Recent data suggest that older age and current smoking could be stronger risk factors for oral HPV persistence than the acquisition [5],[8].

Data reported on the incidence and clearance rates of oral high-risk HPV among (HIV-infected) MSM are scarce, especially those describing the effect of HIV-infection [7],[11],[12]. As HIV-infected MSM are at increased risk of HPV infection and HPV-related head and neck cancer [13],[14], our aim was to compare the 12-month incidence and clearance of oral high-risk HPV infection between HIV-infected and HIV-negative MSM participating in the HIV & HPV in MSM (H2M) cohort in Amsterdam, the Netherlands. In addition, we aimed to assess determinants for oral high-risk HPV incidence and clearance.

In an earlier paper regarding the H2M cohort, we reported 6-month incidence and clearance data of oral HPV infections [15], but we were not able to estimate incidence rates because of limited follow-up time. In the current report covering a longer follow-up period with three measurements per participant, we were able to estimate incidence rates of individual high-risk HPV types, and to assess the effect of HIV infection on time to infection. We also studied clearance using a more rigorous definition (requiring two negative sample results after a positive baseline result rather than just one) than was possible in our earlier paper.

## Methods

### Ethics statement

The Medical Ethics Committee of the Academic Medical Center (AMC) Amsterdam approved this study, and all participants provided written informed consent prior to participation.

### Study participants and sample collection

The H2M study design and sample collection methods have been described previously [4],[15]. In brief, HIV-negative and HIV-infected MSM aged 18 years or older were recruited for a prospective cohort study in 2010–2011 in Amsterdam. Data were collected at baseline, and after 6 and 12 months follow-up. At each visit, participants provided oral samples by rinsing and gargling for 30 seconds using 10–15 ml Scope mouthwash (Procter & Gamble, Toronto, Ontario), and completed detailed risk-factor questionnaires. HIV-related data at baseline were obtained from the Dutch HIV Monitoring Foundation’s national HIV patient database.

### HPV DNA genotyping and classification

Processing of the oral samples and HPV DNA genotyping methods have been described before [4]. DNA extraction was performed using the MagNA Pure LC Total Nucleic Acid Isolation Kit (Roche, Mannheim, Germany), and subsequently HPV DNA amplification was performed using the highly sensitive SPF_10_-PCR DEIA/LiPA_25_ system (version 1) [16]. LiPA_25_ allows simultaneous identification of 25 specific mucosal HPV genotypes, of which the following were classified as high-risk types: 16, 18, 31, 33, 35, 39, 45, 51, 52, 56, 58, and 59 [1].

### Statistical analyses

Since participants could have been infected with or be at risk for more than one high-risk HPV type, the main outcomes of interest were time to incidence of oral type-specific high-risk HPV infections, and clearance of type-specific HPV infections. An incident HPV infection was defined as a type-specific positive test result at 6 or 12 months follow-up in a participant who was free of that specific HPV type at baseline. For incidence analyses, we assumed that a new infection occurred at the midpoint between the date of the first detection and the date of the prior negative test result. Incidence rate ratios (IRR) were calculated using Poisson regression. Kaplan-Meier curves were constructed to explore the cumulative probability of type-specific HPV incidence; log-rank tests were used to compare HIV-infected and HIV-negative MSM. We assessed the independent effect of HIV infection on time to incident oral high-risk HPV infection using the Wei-Lin-Weissfeld method, which is basically a Cox model stratified by HPV type, to account for multiple HPV infections per person, while adjusting for within-subject correlations [17]. We also explored determinants associated with incident oral high-risk HPV infection stratified by HIV-status using the Wei-Lin-Weissfeld method. Multivariable analyses could not be performed for HIV-negative MSM due to low numbers of incident infections.

Among participants who tested positive at baseline, type-specific clearance of oral high-risk HPV infections was explored. Clearance was defined as a participant testing negative at two consecutive visits after a prevalent infection at baseline. Persistence was defined as a participant testing positive at two consecutive visits after a prevalent infection at baseline. Participants with a missing visit were excluded from these analyses. To assess the independent effect of HIV infection on clearance, logistic regression analyses were performed, with generalized estimating equations (GEEs) to account for multiple HPV infections per person, assuming an exchangeable correlation structure [9]. Because of low numbers of oral HPV infections at baseline, determinants associated with clearance could not be examined stratified by HIV status.

For both incidence and clearance analyses, variables that were biologically plausible as well as known determinants from the literature were included a priori in the multivariable models. *P*-values were considered statistically significant at *P* < 0.05. All analyses were performed using Stata (version 13.0; Stata Corp, College Station, Texas, USA).

## Results

### Study participants

Of the 795 participants that were enrolled in the H2M study (previously described [18]), 723 were included in the current analyses. Participants for whom data collection was incomplete (i.e., no HPV test results and/or no questionnaire data, n = 72) were excluded. The 72 excluded participants were younger than the 723 included participants (rank-sum *P* = 0.046), but comparable with respect to HIV status. At baseline, the median age was 38 (IQR 34–42) years among 433 HIV-negative MSM and 47 (IQR 40–53) years among 290 HIV-infected MSM (*P* < 0.001). HIV-infected MSM generally showed higher sexual risk behavior, e.g., the median number of lifetime male sex partners was 300 (IQR 100–1000) for HIV-infected MSM versus 100 (IQR 50–400) for HIV-negative MSM (*P* < 0.001). The median follow-up time was 370 days for HIV-negative MSM (IQR 356–384) and 350 days for HIV-infected MSM (IQR 314–373; *P* < 0.001). Among HIV-infected MSM, the median CD4^+^ cell count was 530 cells/mm^3^ (IQR 410–694), the median nadir CD4^+^ cell count was 220 cells/mm^3^ (IQR 170–320), and 79% (192/243) had an undetectable HIV viral load at baseline.

### Oral high-risk HPV incidence

**Figure 1:**
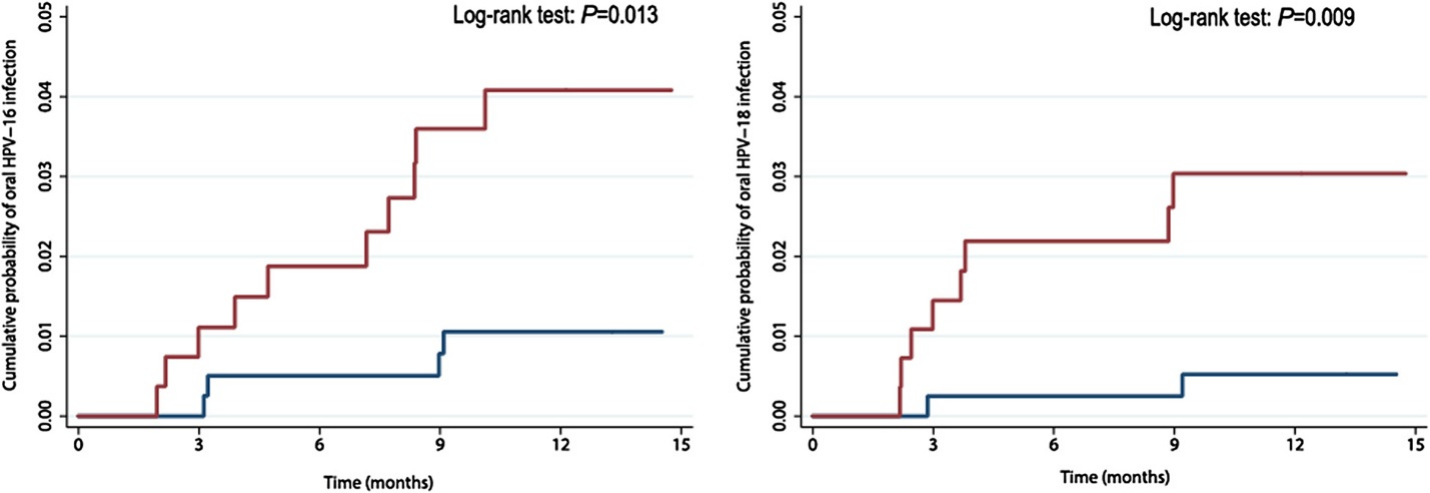
**Cumulative probability of oral HPV-16 and HPV-18 infection.** The cumulative probability of oral HPV-16 and HPV-18 infection in HIV-infected MSM (red lines) and HIV-negative MSM (blue lines) (H2M study, Amsterdam 2010–2012). Abbreviations: MSM = men who have sex with men; H2M = HIV & HPV in MSM.

In total, 114 incident oral high-risk HPV infections were detected. For each HPV type, the incidence rate was higher in HIV-infected than in HIV-negative MSM (Table 1). For HPV-16, the incidence rate was 3.5/1000 person months (PM) in HIV-infected MSM versus 0.9/1000 PM in HIV-negative MSM, with an IRR of 4.1 (95% confidence interval (CI) 1.3-13.2). The incidence rate of HPV-18 was 2.8/1000 PM and 0.4/1000 PM in HIV-infected and HIV-negative MSM respectively (IRR 6.6; 95% CI 1.4-30.8). The 12-month cumulative incidence of HPV-16 was 4.0% in HIV-infected MSM and 1.0% in HIV-negative MSM; for HPV-18 this was 3.0% in HIV-infected MSM and 0.5% in HIV-negative MSM (Figure 1).

**Table 1:** **The number of incident infections** ^**a**^ **, the incidence rate, and incidence rate ratios of individual oral high-risk HPV types in HIV-negative and HIV-infected MSM (H2M study, Amsterdam 2010-2012)**

	HIV-negative MSM				HIV-infected MSM				HIV-infected versus HIV-negative MSM		
	No.	PM	Incidence rate per 1000 PM (95% CI)		No.	PM	Incidence rate per 1000 PM (95% CI)		IRR (95% CI)		*P* -value ^b^
HPV-16	4	4675	0.9	(0.3 - 2.3)	10	2833	3.5	(1.9 - 6.6)	4.1	(1.3 - 13.2)	**0.011**
HPV-18	2	4750	0.4	(0.1 - 1.7)	8	2901	2.8	(1.4 - 5.5)	6.6	(1.4 - 30.8)	**0.007**
HPV-31	1	4782	0.2	(0.03 - 1.5)	7	2918	2.4	(1.1 - 5.0)	11.5	(1.4 - 93.2)	**0.004**
HPV-33	4	4732	0.8	(0.3 - 2.3)	10	2832	3.5	(1.9 - 6.6)	4.2	(1.3 - 13.3)	**0.010**
HPV-35	1	4727	0.2	(0.03 - 1.5)	4	2973	1.3	(0.5 - 3.6)	6.4	(0.7 - 56.9)	0.058
HPV-39	5	4701	1.1	(0.4 - 2.6)	4	2934	1.4	(0.5 - 3.6)	1.3	(0.3 - 4.8)	0.713
HPV-45	2	4775	0.4	(0.1 - 1.7)	4	2973	1.3	(0.5 - 3.6)	3.2	(0.6 - 17.5)	0.161
HPV-51	3	4693	0.6	(0.2 - 2.0)	10	2908	3.4	(1.9 - 6.4)	5.4	(1.5 - 19.5)	**0.005**
HPV-52	3	4743	0.6	(0.2 - 2.0)	8	2938	2.7	(1.4 - 5.4)	4.3	(1.1 - 16.2)	**0.020**
HPV-56	3	4750	0.6	(0.2 - 2.0)	9	2897	3.1	(1.6 - 6.0)	4.9	(1.3 - 18.2)	**0.009**
HPV-58	0	4798	−		7	2972	2.4	(1.1 - 4.9)	−		
HPV-59	1	4770	0.2	(0.03 - 1.5)	4	3001	1.3	(0.5 - 3.6)	6.4	(0.7 - 56.9)	0.058

*Abbreviations:* *MSM* men who have sex with men, *No.* number, *PM* person months, *CI* confidence interval, *IRR* incidence rate ratio.

^a^An incident infection was defined as a positive test result at 6 or 12 months follow-up in a participant who was free of that specific HPV type at baseline.

^b^Significant P values are in bold.

In multivariable analyses, incident oral high-risk HPV infection was significantly associated with HIV infection (adjusted hazard ratio (aHR) 3.8, 95% CI 2.3-6.2) and with a higher number of recent oral sex partners among MSM overall (aHR 2.4; 95% CI 1.4-4.2 for ≥8 compared to ≤2 partners). Among HIV-infected MSM only, a higher number of recent oral sex partners and both recent tobacco smoking and cannabis use were associated with incident oral high-risk HPV infection in multivariable analyses (Table 2). Oral-anal contact in the previous six months was significantly associated with incident oral high-risk HPV infection in univariable analysis, but was not associated in multivariable analyses. We did not find an association of incident oral high-risk HPV infection with age nor with nadir CD4^+^ cell count (Table 2).

**Table 2:** **Univariable and multivariable analyses of determinants of incident oral high-risk HPV infection** ^**a**^ **in HIV-negative MSM and HIV-infected MSM using the Wei-Lin-Weissfeld method (H2M study, Amsterdam 2010–2012)**

	HIV-infected MSM and HIV-negative MSM						HIV-infected MSM						HIV-negative MSM		
	Univariable			Multivariable			Univariable			Multivariable			Univariable		
	HR	(95% CI)	*P* -value ^b^	aHR	(95% CI)	*P* -value ^b^	HR	(95% CI)	*P* -value ^b^	aHR	(95% CI)	*P* -value ^b^	HR	(95% CI)	*P* -value ^b^
**HIV infection**			**<0.001**			**<0.001**	n.a.						n.a.		
No	1.0			1.0											
Yes	4.7	(3.0 - 7.5)		3.8	(2.3 - 6.2)										
**Age (years) by category** ^**c**^			**<0.001**			0.215			0.715			0.356			0.524
≤ 34	1.0			1.0			1.0			1.0			1.0		
35 - 44	1.4	(0.7 - 2.5)		1.0	(0.6 - 1.8)		0.9	(0.4 - 1.9)		0.7	(0.3 - 1.5)		1.6	(0.7 - 4.0)	
≥ 45	2.7	(1.5 - 4.8)		1.5	(0.8 - 2.6)		1.1	(0.5 - 2.2)		1.1	(0.6 - 2.3)		1.7	(0.6 - 5.1)	
**Tobacco smoking and/or cannabis use in previous 6 months** ^**d**^			**0.002**			0.051			**0.010**			**0.017**			0.650
No	1.0			1.0			1.0			1.0			1.0		
Cannabis use only	2.6	(1.4 - 4.9)		1.5	(0.8 - 2.7)		2.5	(1.3 - 5.1)		1.9	(1.0 - 3.6)		0.9	(0.1 - 6.5)	
Tobacco smoking only	1.9	(1.1 - 3.4)		2.0	(1.2 - 3.4)		2.8	(1.4 - 5.4)		2.7	(1.4 - 5.2)		0.9	(0.3 - 2.8)	
Both cannabis use and tobacco smoking	2.4	(1.4 - 4.1)		1.8	(1.0 - 3.0)		2.1	(1.1 - 4.1)		2.0	(1.0 - 3.8)		1.7	(0.7 - 4.1)	
**Oral-anal contact in previous 6 months (rimming)**			0.110			0.530			**0.036**			0.392			0.615
No	1.0			1.0			1.0			1.0			1.0		
Yes	1.4	(0.9 - 2.2)		1.2	(0.7 - 1.9)		1.7	(1.0 - 2.8)		1.3	(0.7 - 2.5)		1.2	(0.5 - 2.8)	
**Number of oral sex partners in previous 6 months by category**			**<0.001**			**<0.001**			**0.004**			**0.004**			**0.024**
≤ 2	1.0			1.0			1.0			1.0			1.0		
3 - 7	0.9	(0.5 - 1.7)		1.0	(0.5 - 1.9)		0.8	(0.4 - 1.7)		0.8	(0.4 - 1.7)		2.0	(0.6 - 6.9)	
≥ 8	2.4	(1.4 - 4.0)		2.4	(1.4 - 4.2)		2.1	(1.2 - 3.7)		2.2	(1.1 - 4.4)		4.3	(1.4 - 13.5)	
**Nadir CD4 cell count (cells/mm** ^**3**^ **) by category** ^**c**^	n.a.								0.108			0.115	n.a.		
≤ 199							1.0			1.0					
200 - 350							0.6	(0.3 - 1.1)		0.6	(0.3 - 1.0)				
≥ 350							1.2	(0.6 - 2.3)		0.9	(0.4 - 1.9)				

*Abbreviations:* *MSM* men who have sex with men, *HR* hazard ratio, *CI* confidence interval, *aHR* adjusted hazard ratio, *n.a.* not applicable.

^a^An incident infection was defined as a positive test result at 6 or 12 months follow-up in a participant who was free of that specific HPV type at baseline.

^b^Based on Wald test. Significant P values are in bold.

^c^As collected at baseline. All behavioral variables were based on questions asked during the follow-up visits; HIV infection status was time-updated.

^d^As there was interaction between tobacco smoking and cannabis use, we created a combined variable. In case of missing values for tobacco smoking, baseline smoking status was carried forward. The route of administration was not specified for cannabis use.

### Oral high-risk HPV clearance

At baseline, 111 oral high-risk HPV infections were observed, of which 59 (53.2%) were cleared, 25 (22.5%) persisted, and 27 (24.3%) showed other patterns (Table 3). After 12 months, 38.5% (n = 5/13) and 55.6% (n = 5/9) of the oral HPV-16 infections were cleared in HIV-infected MSM and HIV-negative MSM respectively.

**Table 3:** **Numbers and percentages (with 95% CI) of cleared oral high-risk HPV infections in HIV-negative and HIV-infected MSM** ^**a**^ **(H2M study, Amsterdam 2010–2012)**

	12-month clearance of oral high-risk HPV infections						
	HIV-negative MSM			HIV-infected MSM			
	No.	%	95% CI	No.	%	95% CI	*P* -value ^b^
HPV-16	5/9	55.6	(21.2 - 86.3)	5/13	38.5	(13.9 - 68.4)	0.666
HPV-18	2/3	66.7	(9.4 - 99.2)	3/6	50.0	(11.8 - 88.2)	1.000
HPV-31	1/1	100.0	(2.5 - 100.0)^c^	5/7	71.4	(29.0 - 96.3)	1.000
HPV-33	1/3	33.3	(0.8 - 90.6)	1/12	8.3	(0.2 - 38.5)	0.371
HPV-35	1/5	20.0	(0.5 - 71.6)	1/3	33.3	(0.8 - 90.6)	1.000
HPV-39	4/4	100.0	(39.8 - 100.0)^c^	2/6	33.3	(4.3 - 77.7)	0.076
HPV-45	0/0	-	-	5/5	100.0	(47.8 -100.0)^c^	-
HPV-51	5/6	83.3	(35.9 - 99.6)	3/5	60.0	(14.7 - 94.7)	0.545
HPV-52	3/3	100.0	(29.2 - 100.0)^c^	3/4	75.0	(19.4 - 99.4)	1.000
HPV-56	1/2	50.0	(1.3 - 98.7)	5/9	55.6	(21.2 - 86.3)	1.000
HPV-58	0/0	-	-	1/2	50.0	(1.3 - 98.7)	-
HPV-59	2/2	100.0	(15.8 - 100.0)^c^	0/1	0.0	(0.0 -97.5)^c^	0.333

*Abbreviations:* *MSM* men who have sex with men, *No.* number, *CI* confidence interval.

^a^Clearance was defined as two consecutive negative results after a positive test result at baseline (1-0-0), persistence was defined as two consecutive positive results after a positive test result at baseline (1-1-1), other infection patterns (1-0-1 or 1-1-0) did not meet criteria for either clearance or persistence.

^b^Based on Fisher’s exact test.

^c^One-sided: 97.5% CI.

HIV status was borderline significantly associated with clearance of HPV infection in univariable analysis, but was not associated in multivariable analyses (aOR 0.5; 95% CI 0.2-1.3; Table 4). In multivariable analyses, a higher number of recent oral sex partners was significantly and positively associated with clearance of high-risk HPV infection (adjusted odds ratio (aOR) 3.4; 95% CI 1.3-9.0 for ≥8 compared to ≤2 partners). Neither age nor tobacco smoking were associated with oral high-risk HPV clearance in uni- and multivariable analyses.

**Table 4:** **Univariable and multivariable analyses of determinants of clearance** ^**a**^ **of 111 oral high-risk HPV infections in MSM using logistic regression analyses with generalized estimating equations (H2M study, Amsterdam 2010–2012)**

	Univariable				Multivariable	
	OR	(95% CI)	*P* -value ^b^	aOR	(95% CI)	*P* -value ^b^
**HIV infection**			**0.049**			0.167
No	1.0			1.0		
Yes	0.4	(0.2 - 1.0)		0.5	(0.2 - 1.3)	
**Age (years) by category** ^**c**^			0.233			0.494
≤ 34	1.0			1.0		
35 - 44	0.5	(0.1 - 2.6)		0.3	(0.1 - 2.0)	
≥ 45	0.3	(0.1 - 1.6)		0.4	(0.1 - 2.3)	
**Current tobacco smoking** ^**d**^			0.696			0.910
No	1.0			1.0		
Yes	0.9	(0.4 - 1.9)		1.1	(0.4 - 2.6)	
**Number of oral sex partners last 6 months by category**			**0.018**			**0.030**
≤ 2	1.0			1.0		
3 - 7	1.4	(0.5 - 3.9)		1.2	(0.4 - 3.6)	
≥ 8	3.6	(1.4 - 9.0)		3.4	(1.3 - 9.0)	

*Abbreviations:* *MSM* men who have sex with men, *OR* odds ratio, *aOR* adjusted odds ratio, *CI* confidence interval.

^a^Clearance was defined as two consecutive negative results after a positive test result at baseline (1-0-0).

^b^Based on Wald test. Significant P values are in bold.

^c^As collected at baseline. All other variables were based on data collected at the 6-month follow-up visit.

^d^In case of missing values at the 6-month follow-up visit, baseline smoking status was used.

## Discussion

In our cohort of HIV-negative and HIV-infected MSM, we found that HIV infection was an independent determinant for oral high-risk HPV incidence, and that around half of the oral HPV infections were cleared within 12 months.

The type-specific incidence rates among HIV-negative MSM (e.g. HPV-16: 0.9/1000 PM) were generally comparable to or somewhat higher than incidence rates reported in a large multinational study among HIV-negative, mainly heterosexual men (HPV-16: 0.8/1000 PM) [5], but the incidence rates among HIV-infected MSM in our study were substantially higher (HPV-16: 3.5/1000 PM). This indicates that HIV infection may be an important determinant for oral HPV incidence. This was even the case after adjustment for age, smoking, oral-anal contact and the number of recent oral sex partners. A higher number of recent oral sex partners was also significantly associated with oral HPV incidence, which is in line with previous reports suggesting that oral sexual behavior may be a risk factor for both oral HPV prevalence [3],[9] and HPV-related head and neck cancer [19].

Although oral high-risk HPV clearance was generally lower among HIV-infected MSM, HIV infection was not a significant determinant for clearance in multivariable analyses. Strikingly, a higher number of recent oral sex partners was significantly associated with *increased* oral HPV clearance. We hypothesize that newly acquired HPV infections (which may be more common among participants with more recent oral sex partners) are more likely to clear within one year, as compared to long-standing persistent infections [6]. This finding could also be explained by the fact that our method of HPV detection was based on the detection of HPV DNA which is not necessarily indicative of an active infection. Therefore, the higher clearance rate in those with a higher number of recent oral sex partners might be because part of the oral HPV in this group could be detections rather than actual active infections.

The current study builds on data previously published, in which the 6-month oral high-risk HPV incidence and persistence were analyzed within the same cohort [15]. Most determinants identified in this report were comparable to those in our earlier report, notably the association between HIV infection and oral HPV incidence. However, in the earlier report an increasing number of recent oral sex partners was borderline significantly associated with oral high-risk HPV incidence in multivariable analyses, while in this report it was a strong and independent determinant. Moreover, while in our previous report no determinants for clearance were identified, we now observed an independent association with the number of recent oral sex partners. Although the study population, sampling and laboratory methods, and research questions were largely the same, the current study adds important information: 1) the follow-up time was doubled, which increased the power of the study and enabled us to assess incidence rates, which facilitates comparisons across studies, and 2) we used a stricter definition of clearance (requiring two negative samples after a positive sample).

This study has some limitations. First, despite the increased follow-up time, the current study still had limited power, especially in the analyses of HPV clearance in HIV-negative MSM. Second, it has been reported that oral HPV genotypes might be missed with a single oral rinse sample and, that this sampling technique could be improved by prior epithelial abrasion or by using multiple sampling methods [20],[21]. Although this potential error could have impact on the accuracy of the incidence and clearance rates, it would not have affected the difference in the incidence or clearance rates between risk groups because the error would be similar for each group. Finally, our analysis of clearance was based on prevalent oral infections: the duration of the infection before enrollment was unknown. Long-term follow-up studies are needed to further assess the natural history or oral HPV infection. Future research could also investigate the role of oral health in acquisition and persistence of oral high-risk HPV infections as recent studies indicated that poor oral health is an independent risk factor for oral HPV infection [22],[23].

## Conclusions

In conclusion, oral high-risk HPV incidence was independently associated with HIV infection and a higher number of recent oral sex partners, while just over half of the prevalent infections were cleared within 12 months. As the incidence of HPV-related head and neck cancer is increasing in many parts of the world [24]-[26], better understanding of the natural history of oral HPV infection is essential for prevention of these HPV-related cancers, especially among high-risk groups such as HIV-infected MSM.

## Authors’ original submitted files for images

Below are the links to the authors’ original submitted files for images. Authors’ original file for figure 1

## Abbreviations

HPV: Human Papillomavirus
MSM: Men who have sex with men
H2M: HIV & HPV in MSM
IRR: Incidence rate ratio
GEEs: Generalized estimating equations
IQR: Interquartile range
PM: Person months
CI: Confidence interval
HR: Hazard ratio
aHR: Adjusted hazard ratio
OR: Odds ratio
aOR: Adjusted odds ratio
