# Supplementary figures and images for: Twelve-month incidence and clearance of oral HPV infection in HIV-negative and HIV-infected men who have sex with men: the H2M cohort study
Source: BMC Infect Dis. 2014 Dec 31;14:668. doi: 10.1186/s12879-014-0668-z (PMC4299566; doi:10.1186/s12879-014-0668-z)

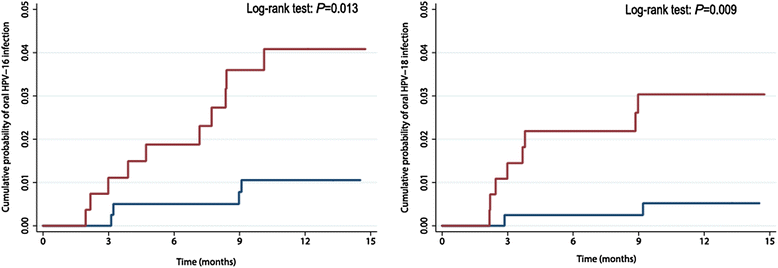

Supplement: Supplementary file 1 — Authors’ original file for figure 1 [file 12879_2014_668_MOESM1_ESM.gif]
